# Supplementary material for: Effects of Primary and Secondary Psychopathy on Deontological and Utilitarian Response Tendencies: The Mediator Role of Alexithymia
Source: Healthcare (Basel). 2022 Aug 29;10(9):1650. doi: 10.3390/healthcare10091650 (PMC9498413; doi:10.3390/healthcare10091650)
Supplement: Supplementary file 1 [file healthcare-10-01650-s001.zip › healthcare-1841171-supplementary.pdf]

Effects of primary and secondary psychopathy on deontological and utilitarian response tendencies: The mediator role of alexithymia

Table S1 The number of participants in each age group

Age	Number of people	Percentage
17.00	63	5.4
18.00	461	39.4
19.00	351	30.0
20.00	209	17.9
21.00	61	5.2
22.00	17	1.5
23.00	4	0.3
24.00	4	0.3

Table S2 English version of the congruent and incongruent moral dilemmas.

	Incongruent Dilemma Variant	Congruent Dilemma Variant
Crying baby	It is war time. Enemy soldiers have taken over your village. They have orders to kill all remaining civilians. You and some of your townspeople have sought refuge in the cellar of a large house. Outside you hear the voices of soldiers who have come to search the house for valuables. A baby with no parents begins to cry loudly. You cover her mouth to block the sound. If you remove your hand from the baby's mouth her crying will summon the attention of the soldiers who will kill you and the others hiding out in the cellar. To save yourself and the others you must smother the child to death. Is it appropriate for	It is war time. Enemy soldiers have taken over your village. They have orders to capture all remaining civilians to make them work quarrying stone in a mine. You and some of your townspeople have sought refuge in the cellar of a large house. Outside you hear the voices of soldiers who have come to search the house for valuables. A baby with no parents begins to cry loudly. You cover her mouth to block the sound. If you remove your hand from her mouth the crying will summon the attention of the soldiers who will capture you and the others hiding out in the cellar. To save yourself and the others from laboring in the mine you must smother the child to death. Is it

	you to smother the child in order to save yourself and the other townspeople from being killed?	appropriate for you to smother the child in order to save yourself and the other townspeople from being captured?
Abortion	You are a surgeon. A young woman you know becomes pregnant, but her body reacts in an unusual fashion. She develops a severe case of preeclampsia, a dangerous syndrome that leads to rapid increases in blood pressure. The only treatment is to deliver the baby. Unless the baby is delivered soon, the mother will die. However, the baby is too young to survive on its own. If it is delivered, it will die. So, although it is very difficult for her, the mother asks you to abort the baby. Is it appropriate for you to perform an abortion in order to save the mother's life?	You are a surgeon. A young woman you know becomes pregnant, but she is not yet ready for children. She has not finished high school, has no income, and was abandoned by the father. If she has the baby now, she will be stuck as a single mother on welfare for the rest of her life. This will make things very hard on her and the baby. She thinks that it would be smarter to wait and have children later. So, although it is very difficult for her, she asks you to abort the baby. Is it appropriate for you to perform an abortion in order to let the mother live a better life?
Vaccine policy	You are a doctor in a health clinic overrun by patients with a serious disease. You just received a shipment of drugs that can cure the disease but the drugs have their own severe side effects. If you administer the drugs to your patients, a small number will die from the side effects but most will live. If you do not, most will die from the disease. Is it appropriate for you to administer the drug to your patients?	You are a doctor in a health clinic overrun by patients with the latest flu virus. You just received a shipment of drugs that can cure the flu but the drugs have their own severe side-effects. If you administer the drugs to your patients, a small number will die from the side effects but most will live. If you do not, most will continue to suffer from the effects of the flu virus for some time. Is it appropriate for you to administer the drug to your patients?
Animal research	You have been hired by a pharmaceutical company to conduct research on their products. Since products must be fit for human use, they are first tried out on animals. Your job is to find out the effects various chemicals have on rats, pigeons, rabbits, and monkeys. Most chemicals have only minor effects on the animals, but some cause them discomfort or even permanent damage. The chemicals you are researching are slated to form part of a new AIDS drug cocktail that will give new hope to millions of AIDS victims around the world.	You have been hired by a pharmaceutical company to conduct research on their products. Since products must be fit for human use, they are first tried out on animals. Your job is to find out the effects various chemicals have on rats, pigeons, rabbits, and monkeys. Most chemicals have only minor effects on the animals, but some cause them discomfort or even permanent damage. The chemicals you are researching are slated to form part of a new acne facial cleanser that will give new hope to people with pimples and greasy skin. You anticipate making many people feel better about their appearance with the

	You anticipate saving many lives with the chemicals. Is it appropriate to test these chemicals on animals?	chemicals. Is it appropriate to test these chemicals on animals?
Torture	You are a police officer, and have recently caught a criminal you have been hunting for some time. He is allegedly responsible for rigging a series of explosive devices: some that have already gone off and some that have yet to detonate. He places explosives outside city cafes and sets them to go off at a time when people are drinking coffee on the patios. In this manner, he has injured many people and might injure many more. Now that the criminal is in custody, you want to know where the unexploded bombs are so you can defuse them. He refuses to talk, so you decide to use "aggressive interrogation techniques" like holding his head under water and beating him. Is it appropriate for you to use "aggressive interrogation techniques" in order to find and defuse the unexploded bombs?	You are a police officer, and have recently caught a criminal you have been hunting for some time. He is allegedly responsible for rigging a series of explosive devices: some that have already gone off and some that have yet to detonate. He places explosives outside city cafes and sets them to go off at a time when no one is around. His explosives are inside paint cans so that they spray nearby objects with paint. In this manner, he has sprayed many cafes with paint and might spray many more. Now that the criminal is in custody, you want to know where the unexploded bombs are so you can defuse them. He refuses to talk, so you decide to use "aggressive interrogation techniques" like holding his head under water and beating him. Is it appropriate for you to use "aggressive interrogation techniques" in order to find and defuse the unexploded bombs?
Hard Times	You are the head of a poor household in a developing country. Your crops have failed for the second year in a row, and it appears that you have no way to feed your family. Your sons, ages eight and ten, are too young to go off to the city where there are jobs, but your daughter could fare better. You know a man from your village who lives in the city and who makes sexually explicit films featuring girls such as your daughter. In front of your daughter, he tells you that in one year of working in his studio your daughter could earn enough money to keep your family fed for several growing seasons. Is it appropriate for you to employ your daughter in the pornography industry in order to feed your family	You are the head of a poor household in a developing country. Your sons, ages eight and ten, work on the family farm every day in order to teach them the value of hard work and providing for others. Your daughter, however, cannot work on the farm due to the heavy lifting required. You worry that she will not learn the value of money if she does not work. You know a man from your village who lives in the city and who makes sexually explicit films featuring girls such as your daughter. In front of your daughter, he tells you that in one year of working in his studio your daughter could earn enough money to keep your family fed for several growing seasons. Is it appropriate for you to employ your daughter in the pornography industry in order to teach her the value of money?

Note: The English version of the moral dilemmas are selected from Conway and Gawronski (2013).

Table S3 Chinese version of the incongruent and congruent moral dilemmas.

	不相容困境	相容困境
哭泣的婴儿	敌人占领了你的村庄。他们下令杀死所有的村民。 你和一些村民躲在一个房子的地窖中。你听到士兵来房子里搜刮贵重物品的声音。这时，一个没有父母的幼儿开始大声哭泣，你用手捂住他的嘴来阻挡哭声。如果把手从幼儿的嘴移开，她的哭声会引起敌人的注意，敌人就会杀死你和地窖中的所有人。为了拯救自己和村民，捂死这个婴儿是合适的吗？	敌人占领了你的村庄。他们下令抓捕所有的村民来采矿。你和一些村民躲在一个房子的地窖中。你听到士兵来房子里搜刮贵重物品的声音。这时，一个没有父母的幼儿开始大声哭泣，你用手捂住她的嘴来阻挡哭声。如果把手从幼儿的嘴移开，他的哭声会引起敌人的注意，敌人就会把你和地窖中的村民抓去采矿。为了避免自己和村民被抓去采矿，捂死这个婴儿是合适的吗？
堕胎	你是一名妇产科医生，在你们的法律中是不能堕胎的，现在有一位孕妇来到你的科室。她患上了严重的疾病，这种病很可能会让她死亡，唯一的治疗方法就是堕胎。虽然这个选择对她来说很难抉择，但她请求你卖一些能让婴儿自然流产的药给她。为了挽救她的生命，卖药给她是合适的吗？	你是一名妇产科医生，在你们的法律中是不能堕胎的，现在有一位孕妇来到你的科室，她年龄较小，也没有收入，还被对象遗弃了，自己也没有做好生孩子的准备。如果她生了这个孩子处境将会更糟糕。并且这种情况对她和孩子都不好。她自己也认为将来有条件了再生孩子是更明智的选择。虽然她知道这样做是不对的，但她请求你卖一些能让婴儿流产的药给她。为了让她过上更好的生活，把药卖给她是合适的吗？
疫苗政策	你是一家诊所的医生，诊所内有许多得了疾病的患者。你刚收到一批可以治愈这种疾病的药，但这些药物本身有严重的副作用。如果你给你的病人服用这种药物，虽然少数人会因副作用而死亡，但大多数人能被快速治愈。如果你不让他们服药，大多数人都会死于这种疾病，给患者服用这种药物是合适的吗？	你是一家诊所的医生，诊所内有许多得了疾病的患者。你刚收到一批可以治愈这种疾病的药，但这些药物本身有严重的副作用。如果你给你的病人服用这种药物，少数人会因副作用而死亡，但大多数人能被快速治愈。如果你不让他们服药，大多数人过段时间也会自愈，只是时间相对来说久一点，给患者服用这种药物是合适的吗？
动物实验	你是一位研发药品的医生，这次你研发了一款可能成为治疗艾滋病患者的有效药物，现在世界上还没有有效治疗艾滋病的药物。但是如果需要投入使用的话还得在大量猴子身上进行实验，这种实验可能会对大量猴子产生极大的副作用，甚至死亡，在猴子身上做实验是合适的吗？	你是一位研发药品的医生, 你研发了一款有效治疗痘痘的药丸，这一款药丸比市面上已有的祛痘产品效果会更好一点，但是如果需要投入使用的话还得在大量猴子身上进行实验，这种实验可能会对大量猴子产生极大的副作用，甚至死亡，在猴子身上做实验是合适的吗？
酷刑	你是一名警察，你抓到了一名逃犯。他把炸弹放在咖啡馆外面，当人们在喝咖啡时引爆。通过这种方式已经伤害了许多人并可能	你是一名警察，你抓到了一名逃犯。他把炸弹放在咖啡馆外面的油漆桶内，当没有人的时候引爆炸弹。把许多地方都弄上了油漆，影响了市容，

	伤害更多的人。你想知道剩余炸弹的位置，以便进行拆卸。但他拒绝回答，唯一的办法就是把他小儿子也抓了起来，通过对他儿子实施一定的酷刑然后逼他回答。抓他儿子是合适的吗？	他还有可能会弄脏更多地方。你想知道剩余炸弹的位置，以便进行拆卸。但他拒绝回答。唯一的办法就是把他小儿子也抓了起来，通过对他儿子实施一定的酷刑然后逼他回答是合适的吗？
困难时期	你生病了需要支付大量医药费，但以你的状况来说你无法借到那么多钱。唯一的方法就是把你 19 岁的女儿嫁给一个有钱的人的儿子，只不过他儿子是个智障，他家愿意支付你所有的医药费，直至你的病痊愈。把你的女儿嫁给这个人是合适的吗？	你的大女儿已经 19 岁了，但是她不能理解你作为父母的辛苦，经常和你吵架、甚至大打出手。唯一的方法就是把她嫁人，然后结婚生子体会当父母的辛苦，虽然她并不愿意嫁个这个人，但是你可以决定这件事情，把你的女儿嫁给这个人是合适的吗？

Note: The Chinese version of the moral dilemmas have been modified in content.

Table S4 The descriptive statistics for each dilemma

Dilemma	Frequencies of acceptance	Percentages of acceptance
Incongruent_dilemma_1	503.00	42.99
Incongruent_dilemma_3	814.00	69.57
Incongruent_dilemma_2	118.00	10.09
Congruent_dilemma_6	436.00	37.26
Congruent_dilemma_5	613.00	52.39
Incongruent_dilemma_4	570.00	48.72
Incongruent_dilemma_5	633.00	54.10
Congruent_dilemma_4	285.00	24.36
Congruent_dilemma_2	136.00	11.62
Incongruent_dilemma_6	584.00	49.91
Congruent_dilemma_3	453.00	38.72
Congruent_dilemma_1	347.00	29.66

Multi-group analysis was conducted to examine whether the indirect effect model differed on the basis of gender. Figure S1 shows females and figure S2 shows males. Standardized indirect effects and 95% confidence intervals for females and males are shown in table S5.

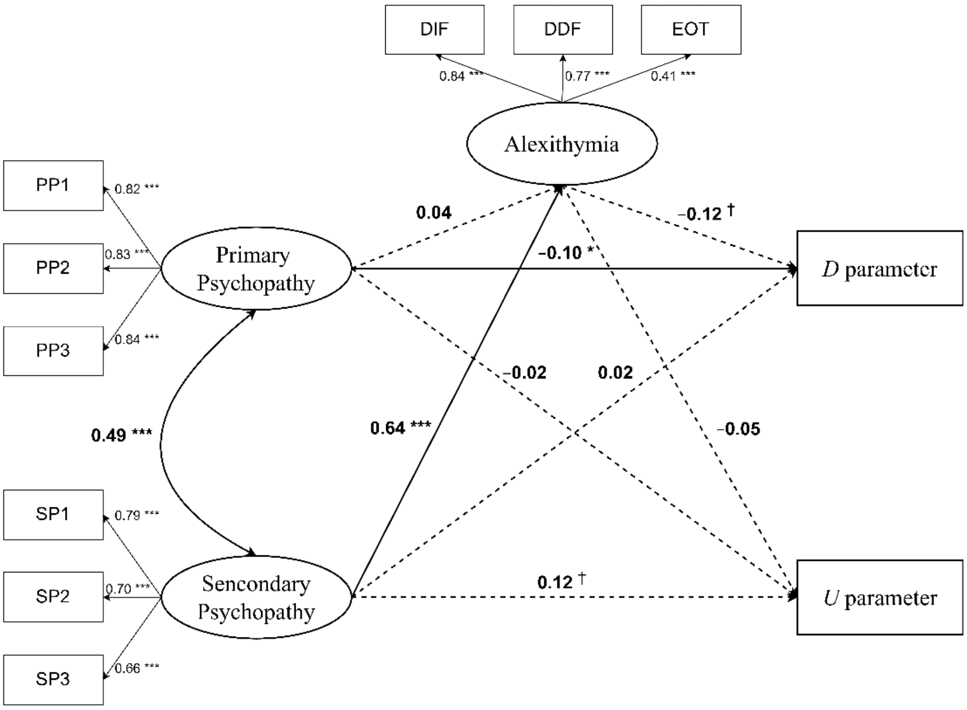

Figure S1. (Females) Mediation model from primary and secondary psychopathy to deontological and utilitarian response tendencies. The standardized coefficients are presented above the arrow. PP1, PP2 and PP3 are parcels of primary psychopathy. SP1, SP2 and SP3 are parcels of secondary psychopathy. DIF, DDF and EOT are dimensions of alexithymia. †p < 0.10; *p < 0.05; ***p < 0.001. N = 703.

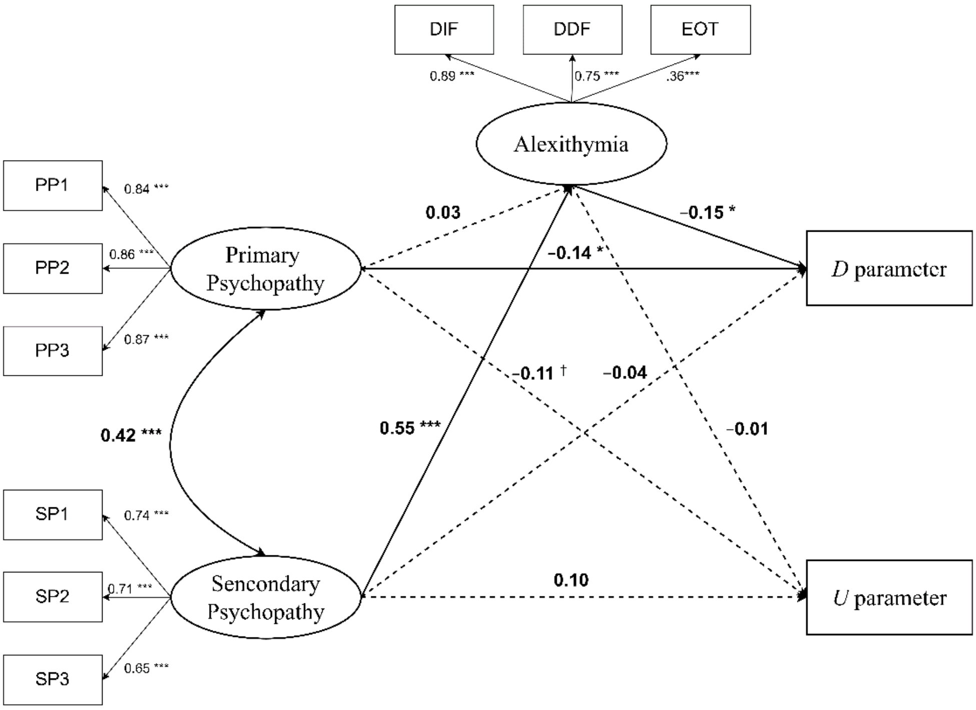

Figure S2. (Males) Mediation model from primary and secondary psychopathy to deontological and utilitarian response tendencies. The standardized coefficients are presented above the arrow. PP1, PP2 and PP3 are parcels of primary psychopathy. SP1, SP2 and SP3 are parcels of secondary psychopathy. DIF, DDF and EOT are dimensions of alexithymia. †$p < 0.10$; *$p < 0.05$; ***$p < 0.001$. $N = 467$.

Table S5 Standardized indirect effects and 95% confidence intervals

Pathways	Females			Males		
	Estimate	Lower	Upper	Estimate	Lower	Upper
1.PP→A→D	−0.004	−0.023	0.005	−0.005	−0.033	0.015
2. PP→A→U	−0.002	−0.018	0.003	0.000	−0.016	0.009
3. SP→A→D	−0.074	−0.162	0.008	−0.082	−0.173	−0.020
4. SP→A→U	−0.032	−0.119	0.049	−0.003	−0.087	0.070

Note: PP = primary psychopathy; SP = secondary psychopathy; A = alexithymia; *U* = *U* parameter (utilitarian response tendencies); *D* = *D* parameter (deontological response tendencies).

Traditional moral judgment (i.e., fewer deontological judgment implies more utilitarian judgment, and vice versa) also can obtain by using process dissociation (PD) approach, which is obtained from the proportion of “acceptable” responses in incongruent dilemmas (Conway & Gawronski, 2013). Higher traditional moral judgment score reflects participants had a strong preference for utilitarian judgment over deontological judgment. It is consistent with previous studies (Conway & Gawronski, 2013), the *U* parameter correlated positively with traditional moral judgment, whereas the *D* parameter correlated negatively with traditional moral judgment. Moreover, psychopathy correlated positively with traditional moral judgment. Furthermore, primary psychopathy and secondary psychopathy correlated positively with traditional moral judgment (see Table S6).

Table S6 Descriptive and Correlations between among the variables (*N* = 1170)

Variables	*M*	*SD*	1	2	3	4	5	6
1.Traditional moral judgment	0.46	0.23	1					
2.*U* parameter	0.14	0.23	0.53***	1				
3.*D* parameter	0.63	0.23	−0.75***	0.11***	1			
4. Psychopathy	52.70	7.72	0.15***	0.01	−0.16***	1		
5.Primary psychopathy	30.87	5.84	0.12***	−0.03	−0.15***	0.92***	1	
6.Secondary psychopathy	21.83	3.29	0.15***	0.06*	−0.12***	0.72***	0.38***	1
7.Alexithymia	49.88	9.64	0.15***	0.01	−0.16***	0.44***	0.29***	0.51***

Note: *U* parameter = utilitarian response tendencies; *D* parameter = deontological response tendencies; *M* = mean; *SD* = standard deviation; *$p < 0.05$; ***$p < 0.001$.

We also examined whether alexithymia mediated the relationships between psychopathy and deontological and utilitarian response tendencies. The bootstrap procedure with 5000 replicates and a 95% confidence interval were adopted to examine the significance levels of indirect models, the age, and gender as control variables. The tested indirect effect model obtained acceptable fit indices ($\chi^2/df = 6.51$, RMSEA = 0.07, CFI = 0.92, TLI = 0.87, SRMR = 0.05), and all the factor loadings were highly significant ($p < 0.001$) (see Figure S3).

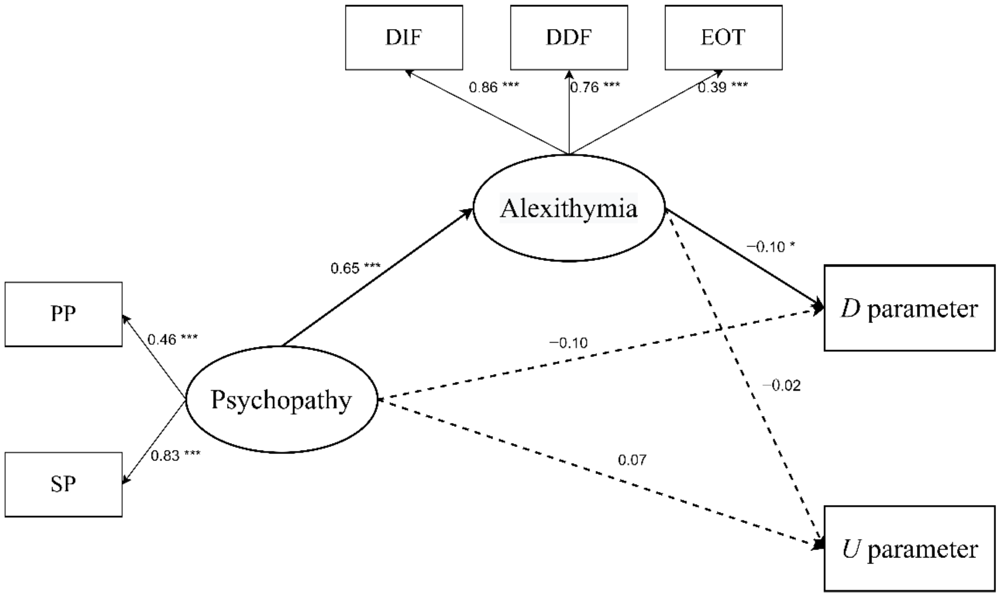

Figure S3. Mediation model from psychopathy to deontological and utilitarian response tendencies. The standardized coefficients are presented above the arrow. PP: primary psychopathy. SP: secondary psychopathy. DIF, DDF and EOT are dimensions of alexithymia. *$p < 0.05$; ***$p < 0.001$.

The mediation analyses results showed that alexithymia (95% CI = [−0.134, −0.004]) played a significant and independent mediating role between psychopathy and deontological response tendencies (see Table S7). Specifically, psychopathy had a highly significant positive effect on alexithymia (β = 0.65, $p < 0.001$), which in turn

had a significant negative effect on deontological response tendencies (β = −0.10, $p < 0.05$).

Table S7 Standardized indirect effects and 95% confidence intervals

Pathways	Estimate	Lower	Upper
1.P→A→D	−0.067	−0.134	−0.004
3. P→A→U	−0.013	−0.081	0.048

Note: P = psychopathy; A = alexithymia; *U* = *U* parameter (utilitarian response tendencies); *D* = *D* parameter (deontological response tendencies).
